# Spontaneous remission of Crohn's disease following a febrile infection: case report and literature review

**DOI:** 10.1186/1471-230X-11-57

**Published:** 2011-05-19

**Authors:** Stephen A Hoption Cann, Johannes P van Netten

**Affiliations:** 1 1School of Population and Public Health, University of British Columbia, Vancouver, BC, V6T 1Z3, Canada

## Abstract

Crohn's disease is a chronic illness that may often follow a relapsing-remitting course. Many of the factors that may be associated with the spontaneous remission of this disease (i.e. not related to specific treatment) remain to be determined. In the present report, we review the medical history of a patient with a long history of moderate to severe Crohn's whose complete remission immediately followed the development of a febrile infection.

The patient first developed symptoms of Crohn's in her late adolescent years. At the time of diagnosis at age 23, she was placed on mesalamine - without effective control her disease symptoms. Due to progressive deterioration, the patient underwent a bowel resection at age 25. Soon afterwards symptoms recurred, gradually increasing in severity. In February 2005, at age 36, the patient developed a painful abscess associated with a rectal fistula. Other symptoms at the time included chronic bone and stomach pain, swollen joints, and debilitating fatigue. Surgical correction was scheduled in mid-March. In late February, the patient developed a respiratory infection associated with fevers of 103-104°F. After the onset of fever, the abscess pain disappeared and this was soon followed by a disappearance of all other disease symptoms. By the time the corrective surgery occurred, she had no Crohn's symptoms. Her remission lasted 10 weeks when the previous symptoms then reappeared. The patient has subsequently used a variety of conventional therapies, but still suffers from severe symptoms of her disease.

In recent years, a growing body of literature has emphasized the important role that innate immunity plays in the etiology of Crohn's disease; however, a key component of innate immunity, the febrile response, has been overlooked. Other cases of spontaneous remission following febrile infection in inflammatory bowel disease have been reported. Moreover, induction of a febrile response was in the past used as a treatment for inflammatory bowel disease, but was later replaced by surgery and corticosteroids. Further exploration of this arm of the innate immune response may provide new opportunities for patients where conventional therapies fail to secure relief.

## Background

Crohn's disease is a chronic inflammatory disease of the gastrointestinal tract that often follows a relapsing-remitting course. Current treatment regimens for Crohn's disease focus on immunosuppressive agents including aminosalicylates, corticosteroids, purine analogues such as azathioprine or 6-mercaptopurine, and biologic therapies such as tumor necrosis factor alpha inhibitors. Along similar lines, surgery is frequently employed to excise regions of immune-mediated inflammatory activity. Contrasting such approaches, recent studies have suggested that underlying defects in innate immunity may play a role in the development of this disease [1,2]. Innate immune deficiencies may in turn leave predisposed individuals vulnerable to an infection or infections that cause the disease. In support of a role for infection, various antibiotic [3] and probiotic [4] regimens have been explored in an attempt to eradicate or displace the suspect pathogen(s). Many pathogens have been implicated with the idea that the disease may result from some form of occult infection [5,6] or from reduced control/decreased biodiversity of commensal bacteria [7,8]. While the probiotic or antibiotic approach may lead to a lessening of inflammation and symptoms, the ability of such regimens to produce cures is limited and antibiotic side effects prevent its long term use.

Although many studies have highlighted defects in the innate immune response, the febrile response arm remains a critical, but often ignored component of innate immunity [9,10]. In the present case study, we report on the temporary remission of severe Crohn's disease following an influenza-like illness with repeated high fevers and review the literature associated with this phenomenon.

## Case presentation

The patient is a 40 year old female with lengthy history of moderate to severe Crohn's disease. The patient's family history included a father who was diagnosed with Crohn's disease at age 20. He later developed colon adenocarcinoma at age 46 years, dying the following year from his cancer.

The patient first developed symptoms of her disease in college at age 18. Symptoms at the time included abdominal pain, weight loss (15 lbs), and debilitating fatigue. However, even previous to this time, the patient suffered for many years from pain in her bones and joints. During her college years, she continued to lose weight. At age 23, following the onset of severe abdominal pain and an inability to eat or drink, a diagnosis of Crohn's disease was finally made. The disease was found to occupy the ileocaecal region. At that time, she began treatment with mesalamine, although it was not effective in controlling her symptoms.

At age 25, the patient presented with symptoms of acute bowel obstruction. Surgery revealed volvulus of the ileocaecal region and 10 inches of the distal ileum and entire caecum were excised. Symptoms recurred fairly soon afterward, but it was a further 6 to 7 years before they became severe.

In February 2005, at age 36, the patient developed a painful fistula-associated rectal abscess. At this time, the patient's ongoing symptoms included chronic bone and abdominal pain, swollen joints, and debilitating fatigue. On February 18th, she was placed on cephalexin to treat the abscess previous to a scheduled surgery to correct the fistula. Following a week with no improvement in abscess pain while on the antibiotic, the patient then developed an influenza-like infection (high fevers, chills, headache, cough, and rhinorrhea). After the first onset of fever, the abscess pain was no longer apparent. The recurrent fevers (103-104°F) from the infection persisted for four days and were associated with a complete disappearance of all other disease symptoms. This was followed by an increase in appetite and weight. On March 18th, the patient underwent the scheduled surgery to correct the fistula. Her energy level had been continually improving and after a month she was running up to 3 miles a day and was able to run up hills that she could not even walk up prior to her remission. After approximately 10 weeks of this symptom-free remission, her previous disease symptoms began to return. The initial symptoms included hugely swollen knees and diarrhea. The patient was again placed on cephalexin in the expectation that it might result in another remission, but the intervention had no effect on symptoms.

Since that remission, the patient subsequently used: budesonide; triple antibiotic therapy; infliximab with prednisone; and is presently on adalimumab with methylprednisolone to treat swollen and painful joints. The patient's current symptoms include bone pain, recurrent painful blisters on her hands, crusting and bleeding of the external nares, oral ulceration, chronic abdominal pain with periodic diarrhea, and swollen and painful joints with occasional extreme pain and swelling.

## Conclusions

In the present case, the patient was diagnosed with Crohn's 13 years before the infection-associated remission, although she was symptomatic with the disease at least 17 years before this remission. The patient's only preceding remission was that following small bowel surgery; otherwise the patient had never been symptom-free and ever-present were symptoms including chronic fatigue, bone and abdominal pain, painful and swollen joints, blisters and ulceration and a poor appetite causing her to be chronically underweight. The patient continues to struggle with these many symptoms which have not been effectively controlled despite intensive medical management.

Lobel and colleagues reported on an interesting series of four Crohn's disease and one ulcerative colitis remission that followed febrile infections [11]. The patients' fevers, usually high, were of two weeks or greater in duration. In four of five cases, no etiology for the fever could be determined, and in the fifth patient, the infection resulted from a perforation of an ileocolic anastomosis and intraabdominal, perihepatic and peripancreatic abscesses. None of the infections were respiratory in nature. There were four females with Crohn's disease (age range 24 to 31 years) and one 34 year old male with ulcerative colitis. Reported remissions were of 2-10 years in duration with no recurrences at the time of reporting, although three subjects remained on maintenance therapy subsequent to their remissions. All patients were taking 6-mercaptopurine (6MP) at the time of remission and the authors hypothesized that fever along with 6MP-induced leukopenia was an important factor in the remissions.

A therapy to stimulate the innate febrile immune response in inflammatory bowel disease dates back to Arthur Hurst, who in 1921 [12] experimented with a dysenteric bacterial serum therapy for one of his patients with ulcerative colitis. Hurst would later report that there was no recurrence when he last saw the patient 15 years later [13]. Hurst, who subsequently treated many more ulcerative colitis patients with this therapy, summarized several of his notable findings [14]. First, he found that intramuscular injections were beneficial, but less frequently than intravenous injections; although he did raise the concern that an anaphylactic reaction may occur following intravenous injection of the vaccine. He further observed that rapid recovery was most likely in the early stages of disease, but could occasionally be very striking in long standing cases. Finally, he noted that recurrence was much reduced if treatment was continued until the sigmoidoscope shows no trace of inflammation, even if symptoms had already disappeared for some weeks.

Based on the preliminary findings of Hurst, in the 1920s and 1930s Burrill Crohn (who first characterized regional ileitis or Crohn's disease [15]) experimented using various intravenous therapies for the treatment of ulcerative colitis. He stated that in approximately 45% of cases there was a persistent cessation of symptoms and restitution to a normal state. Crohn made some interesting observations on the use of Hurst's immunotherapy, which he used regardless of whether or not dysentery organisms could be detected in a patient's blood [16]:

"A severe febrile reaction was welcomed. A certain degree of anaphylactic shock was regarded as more beneficial than deleterious. The most beneficial results were seen in those patients in whom the intravenous injection of serum resulted in immediate serum shock analogous to a non-specific protein reaction and in those who showed late serum sickness with urticaria and even joint manifestations. The use of polyvalent anti-dysentery serum seemed to give us best results.

In order to determine whether the effect of this serum was a specific or a non-specific one, we attempted, in a number of cases, to duplicate the results by the intravenous injection of typhoid vaccine, such representing a convenient and direct method of nonspecific protein therapy. Some fairly good results were seen. Other miscellaneous methods of treatment were tried at various times in the course of years, these methods including autogenous vaccines of fecal organisms, Bargen's serum and vaccine, transfusions, etc., some with good results, many of them without any noticeable effect. It would seem that among the various types of intravenous therapy, no one item seemed to have a specific effect upon the disease. It soon became obvious that any protein agent which would produce a protein shock and a febrile reaction, could bring about a beneficial change in the chronic course of this disease. The change from the slow, lethargic chronicity into a sudden flare-up induced by the protein therapy of whatever type, frequently seemed to alter the long drawn-out course of the malady. After several severe protein shocks, the temperature would frequently subside, diarrhea gradually and more slowly diminish until constipation was achieved and the general health of the patient, appetite, strength and weight began to show steady improvement."

Following World War II, new treatment options became available including antibiotics and corticosteroids. By that time, Crohn had discontinued using bacterial vaccines for his patients with inflammatory bowel disease in preference to surgery, which initially seemed to lead to a very high cure rate: "The percentage of surgical cures is high, even when reckoned conservatively, and it is grossly well over 80 per cent [17]." Although in subsequent years, it became apparent to Crohn that recurrences following surgery were not uncommon, and similarly, other promising therapies failed to meet initial expectations [18].

The possibility exists that other factors may have been responsible for the patient's remission. For example, the antibiotic cephalexin could have played a role, although some facts warrant against such a possibility. The patient had been on cephalexin for many days without any effect on her symptoms, and following disease recurrence the rechallenge with cephalexin had no impact on her symptoms. As to the surgery, by the time the operation for her fistula had taken place, her Crohn's symptoms had already abated and therefore it was an unlikely factor in her remission. In contrast to the case series by Lobel, the acute infections that were associated with remissions were of a longer duration than in the present case; however, they also correspondingly produced remissions of a much longer duration.

In the present case, the complete remission was unusual for this patient due to a long history of severe and poorly controlled disease. Moreover, the remission associated with the patient's febrile infection is analogous to other reports of spontaneous or induced remissions following fever. In fact, the prompt improvement noted in these reports, and in the present case, provides further support for the hypothesis that the innate immune response plays a key role - an adaptive immune response would require a longer time period to develop. Strategies that try to suppress the immune system or those that try to eradicate or displace harmful bacteria have shown limited efficacy. An alternative approach would be to develop therapies that specifically activate the innate febrile immune response, and thereby attempt to reassert immune system control over pathogens or harmful indigenous flora.

## Competing interests

SAHC and JPvN have interests in a company that manufactures a sterile bacterial preparation for the treatment of cancer.

## Authors' contributions

SAHC was in direct contact with the patient. SAHC drafted the manuscript with the assistance of JPvN. SAHC and JPvN made the final corrections and comments. Both authors read and approved the final manuscript.

## Pre-publication history

The pre-publication history for this paper can be accessed here:

http://www.biomedcentral.com/1471-230X/11/57/prepub
